# Rapid Return for School Refusal: A School-Based Approach Applied With Japanese Adolescents

**DOI:** 10.3389/fpsyg.2019.02862

**Published:** 2019-12-20

**Authors:** Naoki Maeda, David Heyne

**Affiliations:** ^1^School of Social Welfare, Kyushu University of Health and Welfare, Nobeoka, Japan; ^2^Institute of Psychology, Leiden University, Leiden, Netherlands

**Keywords:** school refusal, school-based intervention, rapid school return, flooding, Japanese school-refusing adolescents

## Abstract

Cognitive behavioral therapy (CBT) is often effective in the treatment of school refusal (SR). Its usefulness is limited, however, if youth displaying SR also refuse to attend treatment sessions. In these cases parents and school staff may consider using school-based interventions that do not rely on face-to-face assessment and treatment with the young person. The current study examined the effectiveness of a school-based intervention applied in Japan to achieve rapid return to school among adolescents displaying SR. Between 2009 and 2015, the parents of 62 adolescents displaying SR were invited to implement a school-based rapid return approach. Thirty-nine parents agreed to implement the approach and 23 decided to wait until their child spontaneously attended school. Of the 39 cases in which the approach was implemented, 28 adolescents (72%) resumed attendance at their original school, 2 (5%) transferred to another school, and 9 (23%) did not resume attendance. In contrast, all 23 non-intervention cases continued to refuse to attend school for 3 months or longer, and none of these adolescents returned to regular school attendance within 9 months. This study tentatively suggests that the rapid return approach may be an effective form of intervention for adolescents displaying SR and simultaneously refusing to attend individual therapy. Because this approach is ethically complex, involving forced school attendance in adolescence, it should only be employed under specific circumstances. These circumstances are discussed.

## Introduction

### School Refusal

School refusal (SR) is said to occur when a child or adolescent shows reluctance or refusal to attend school in association with emotional distress (Heyne et al., 2019). Commonly used criteria for classifying SR are those originally proposed by Berg et al. (1969) and reformulated by Berg (1997, 2002): (a) remaining at home with the knowledge of the parents; (b) an absence of severe antisocial behavior, apart from possible aggressiveness when the young person is forced to go to school; (c) parental attempts to get the child to attend school; and (d) displaying emotional upset at the prospect of attending school. SR occurs among 1–2 percent of the population of school-aged youth^1^ (Heyne and King, 2004) and the peak age of onset is in early adolescence (Heyne et al., 2014).

SR is regarded as a serious problem in the fields of medicine, welfare, and education (Nishida et al., 2004) with negative short- and long-term consequences (Maynard et al., 2018). Short-term consequences include poor academic performance, family difficulties, and worsening peer relationships, while long-term consequences include academic underachievement, employment difficulties, and increased risk of psychiatric illness (Fremont, 2003; Sewell, 2008; Heyne et al., 2011). Without appropriate intervention SR may be prolonged and become more difficult to treat (Glaser, 1959; Hersov, 1972; King et al., 1998; Okuyama et al., 1999; Sonoda et al., 2008). The prolongation of SR increases the youth's anxiety about school return (Warnecke, 1964; Terada, 2015) and likely reduces their motivation for resolving an aversion to attending school.

School attendance problems (SAPs) like SR are a major concern in compulsory education in Japan, where youth are required to attend 6 years of elementary school (ages 7–12 years) and 3 years of junior high school (ages 13–15 years). The Japanese Ministry of Education, Culture, Sports, Science and Technology (Ministry of Education, Culture, Sports, Science, and Technology-Japan (MEXT), 2003) defines SAPs as absence from school or inability to attend school on more than 30 full days a year, due to physical, psychological, social, and/or emotional factors, excluding cases involving medical and economic reasons for absence. According to the Japanese government (Ministry of Education, Culture, Sports, Science, and Technology-Japan (MEXT), 2017) the number of elementary school students displaying a SAP is ~35,000 (0.5% of elementary school students), the highest rate since data collection began in 1991. Among junior high school students the number is ~110,000 (3.3% of junior high school students), also the highest rate since 1991. In addition, there are many youth who visit the school nurse's office during the school day or attend adaptation classes and some of these would fulfill partial criteria for SR (Maeda, 2016).

### Clinic-Based Psychosocial Treatment for School Refusal

In the last 20 years, studies of treatment for SR have focused mainly on cognitive behavioral therapy (CBT; e.g., King et al., 1998; Last et al., 1998; Ollendick and King, 1999; Bernstein et al., 2001; Heyne et al., 2002), confirmed by a recent meta-analysis (Maynard et al., 2018). The main goal of CBT for SR is to help youth resume a normal developmental pathway via a reduction in emotional distress and a return to regular school attendance (Heyne and Sauter, 2013). Common CBT interventions applied with youth are psychoeducation, relaxation training, social skills training, cognitive therapy, and exposure (Heyne, 2006). Parents are provided with psychoeducation and supported in the use of behavior management strategies (e.g., instruction-giving; planned ignoring; positive reinforcement) aimed at helping the young person attend school (Heyne, 2006).

Graded exposure to school attendance is prominent in five manualized CBT interventions for SR (Heyne et al., 2015). In some cases exposure is not graduated (e.g., Kearney and Albano, 2007) and emphasis is placed upon parents forcing their child to attend school full-time, consistent with the behavioral technique of flooding. Evidence for the efficacy of flooding in cases of SR is lacking (Elliott and Place, 2019) and clinical opinion varies considerably regarding how quickly the young person should return to school and the role of parents in getting a child to school (Heyne and Sauter, 2013).

Conceptual and terminological ambiguity may have clouded discussion of these topics. For example, some of the terms used to refer to the general time-frame for return to school are “early return” (Kennedy, 1965), “immediate return vs. later return,” and “much later school return” (Berecz, 1969). The process for achieving school return has been referred to as “rapid return” (Leventhal et al., 1967), “rapid treatment” (Kennedy, 1965; King and Ollendick, 1989), “forced school attendance” (Hersen, 1971; Gullone and King, 1991; Kearney, 2003), “*in vivo* flooding” (Blagg and Yule, 1984; Blagg, 1987), “rapid vs. graduated re-entry” (King and Ollendick, 1989), and “the escorting process” (Heyne and Rollings, 2002; Herbert, 2004).

To promote clarity, Heyne and Sauter (2013) distinguished between “early full-time” and “early part-time” increase in attendance. “Early” refers to an intention for the young person to start increasing attendance within at least 4 weeks of commencing treatment, “full-time” refers to a flooding-based approach (i.e., full-time attendance from the first day of planned school return), and “part-time” refers to a graduated increase (e.g., successive increase in the number of classes attended). Many youth participating in treatment for SR choose a part-time increase in attendance (Heyne and Sauter, 2013) which is held to reduce treatment drop-out (Last and Francis, 1988).

There is no systematic evaluation comparing part-time increase (i.e., graded exposure) and full-time increase (i.e., flooding) for the treatment of SR. An early study of the acceptability and perceived effectiveness of interventions for SR suggested that behavior management by parents, involving forced school return (i.e., flooding), was more acceptable and perceived to be more effective than home tuition with psychotherapy, hospitalization, and medication (Gullone and King, 1991). That is, adolescents, parents, and professionals (teachers and nurses) all rated behavior management as more acceptable and likely to be effective than the other interventions. It should be noted, however, that the respondents were potential but not actual consumers of interventions for SR. Moreover, the case illustration used to exemplify different interventions was based on a child of 6 years and not an adolescent.

Support for interventions emphasizing flooding comes from case studies and non-randomized trials reported prior to the 1990s. For example, Leventhal et al. (1967) reported two cases that involved prompt school return implemented by parents. Both youth (a 9-year-old girl and a 15-year-old boy) returned to school when the parents abandoned a passive approach in favor of forced school attendance. The authors mentioned that early return was necessary to prevent the youths' anxiety becoming entrenched. Baideme et al. (1979) reported the case of a 9-year-old girl who was firmly escorted to school by her parents. The authors, Adlerian family therapists, insisted that quickly returning youth to school is important, irrespective of the etiology of SR symptoms. Earlier, Rodriguez et al. (1959) qualified that the sole exception to firmly insisting on early return to school is in the case of the “overtly psychotic child” (p. 544).

The first treatment series which focused on a “rapid treatment program” was reported by Kennedy (1965). Fifty youth aged 4–16 years (80% aged 12 or younger) fulfilled the following criteria for Type I SR: first episode (100% of the youth); Monday onset following illness previous Thursday or Friday (98%); acute onset (96%); grade six and below (80%); death theme present (88%); mother's health an issue (88%); marital harmony (94%); good parental mental health (94%); father helps in household management (84%); and parents achieve insight quickly (98%). Kennedy's intervention encompassed a structured interview with parents and a brief interview with the young person. Parents were introduced to a plan incorporating advice about: not discussing the child's problem at length; the father taking the child to school, avoiding discussion or questions about the child's symptoms; leaving school promptly after dropping the child off; and socially and tangibly reinforcing the child for attendance. Forced attendance involved a willingness to use any force necessary, but it was usually sufficient that parents were convinced of the necessity of this action and were decisive. The plan also involved school staff keeping the child at school and restricting the mother's presence at school. In a brief interaction with the young person the therapist taught him or her about the importance of facing fears, using analogies (e.g., getting straight back on a horse after falling off) as well as personal examples. All 50 cases showed complete remission of SR symptoms within 3 days. No diagnostic evaluations were conducted, but Kennedy reported no evidence of SR or symptom substitution during 8 years of follow-up. The fact that all youth aged 12 and older (*n* = 13) were successfully treated signals the possibility of applying a flooding-based approach with adolescents displaying SR. At the same time, the effectiveness of this rapid treatment program is uncertain in the absence of non-treatment controls.

Building on Kennedy's (1965) work, Blagg (1977) developed a more comprehensive behavioral treatment encompassing four principles: (1) desensitization through humor and emotive imagery; (2) blocking avoidance through insistence upon immediate return to full-time attendance during the early stages of treatment and using force if necessary; (3) maximizing positive reinforcement for school attendance both at home and school; and (4) extinction of protests, fear reactions, and psychosomatic complaints through contingency management. *In vivo* flooding in the form of school return “even under escort” was applied when certain conditions were met, such as no genuine physical complaints for the young person, enrolment at an appropriate school, a united approach between parents and school staff, the young person has strategies for coping with school return, the school has made arrangements to help the young person settle in, and parents have received detailed advice about how to respond when their child protests (Blagg and Yule, 1984, p. 122). Specific recommendations were provided for finding a suitable escort (e.g., have two escorts when it is expected that the young person will protest strongly; consider involving close relatives when parents lack control or are extremely anxious; involve a teacher, social worker, or psychologist if the family cannot resolve the problem). Parental resistance was addressed by being supportive while confronting parents with the reality of the situation (e.g., secondary factors arise during absence from school; life often requires facing frightening situations). Blagg (1977) considered his intervention suitable for some Type II school refusers (e.g., older youth who displayed earlier episodes of SR).

Blagg and Yule (1984) evaluated this behavioral treatment approach (BTA) by comparing outcomes for 30 youth in the BTA group, 16 youth who were hospitalized (HU), and 20 youth who received home tuition plus psychotherapy (HT). Youth were aged 11–16 years, except for 6 youth (9%) who were younger than 11 (5 of the 6 were in the BTA group). All youth fulfilled SR criteria similar to those proposed by Berg (1997, 2002). The flooding-based BTA was the most economical form of intervention, lasting 2.5 weeks on average, compared with 45.3 weeks for HU and 72.1 weeks for HT. The researchers argued that BTA was also significantly more effective than the two other approaches. An average of 1 year after treatment, successful outcome (i.e., return to full-time schooling without a lapse resulting in absenteeism) was observed for 93% of BTA cases, 38% of HU cases, and 10% of HT cases. Twenty-five of the 30 youth in the BTA group (83%) attended school at least 80% of the time, compared with 5 HU cases (31%) and none of the HT cases. The authors contended that anxiety experienced by youth or caregivers as a result of the BTA rapid approach was more than justified on account of the remarkably quick adjustments made by most children.

On the face of it, it seems that taking pressure off the young person to attend school is not an effective intervention. This perspective is shared by Berg (1985) and supported by the King et al. (1998) randomized controlled trial of CBT for SR. In the King et al. study, 88% of youth (5–15 years) whose parents received guidance in enforcing school attendance showed a significant improvement in school attendance, compared with 29% of youth in families placed on a wait-list. King et al. also noted that the youths' participation in CBT may have helped them prepare for school return, but there were still some youth who showed reluctance or procrastination, and it is likely “that parents then played an invaluable role in prompting school attendance and escorting the child to school in a firm manner” (p. 402). The Blagg and Yule (1984) evaluation of BTA also suggests that a firm approach by parents is valuable, but conclusions based on their study need to be tempered by two main considerations. First, allocation to the three treatments was not randomized so it could be argued that easier cases were treated with BTA. Second, youth in the flooding-based BTA were significantly younger than those in the HU group.

### Clinic-Based Psychosocial Treatment for School Refusal in Japan

There appear to be two main approaches to clinic-based treatment for SR in Japan. The first is a passive approach whereby pressure to return to school is removed, with an expectation of spontaneous recovery (Honjo, 1990; Kawai, 2003; Kawai and Sakurai, 2003). The goal of treatment is not the resumption of school attendance (Nakagawa, 1998; Saito, 2007; Meguro, 2009) but the development of the individual's self-concept via counseling (Tabata, 1980; Fukaya, 1983). The other approach can be classified as an active approach aimed at returning the young person to school as soon as possible.

One example of the active approach is found in Sonoda's (1971) report of a clinic-based behavioral intervention for youth (7–19 years) who displayed SR according to Berg et al. (1969) criteria. Forced school attendance by parents and clinic staff was utilized in 15 of 23 cases (8 youth at elementary school, 2 at junior high school, and 5 at high school). For the 8 other cases (1 at junior high, 6 at high school, and 1 at university), behavioral counseling sessions were implemented with the young person and their parents. Youth sessions focused on modifying cognitive distortions about school life. In parent sessions the emphasis was placed on the importance of blocking the youth's avoidance of school. School attendance records were utilized as an outcome measure. Fourteen of the 15 youth in the forced school attendance group returned to regular school attendance, as did five of the eight youth in the behavioral counseling group. Sonoda (1971) concluded that the flooding-based school return approach implemented by parents (mainly fathers) was highly effective for SR. This is one of the few studies reporting on forced school attendance for adolescents as old as 17 (*n* = 4). Because the study was uncontrolled, the benefit of forced school attendance relative to other treatment approaches is unknown.

Another example of the active approach is found in the work of Aida (1978). Aida hypothesized that SR among adolescents was maintained by fathers allowing the adolescent to be absent from school, regardless of the cause of SR. Fathers were thus encouraged to block the avoidance of school by using forced school attendance. Improved attendance was observed among five out of six cases of adolescent SR (12–17 years). Aida suggested that paternal blocking of school avoidance is effective for adolescent SR, adding that forced school attendance should not be used when adolescents have a mental disorder.

### School-Based Behavioral Intervention for School Refusal in Japan

In rural areas of Japan there seem to be few specialized institutions that provide clinic-based psychosocial interventions resembling the active approach to treatment for SR. To illustrate, Maeda (2011) interviewed the parents of 21 Japanese youth displaying SR. These parents had consulted psychiatrists or child psychologists for treatment at clinic-based institutions in rural areas. All of the parents had been advised to just “wait and see” until such time as their child demonstrated spontaneous school return. In the region where the study was conducted, Japanese clinicians did not employ CBT for SR.

In situations where clinic-based services are not available, or an active approach to SR is not offered, a school-based behavioral intervention may be required. The first author (NM), a part-time school counselor in public junior high schools, developed a school-based support system comprising a rapid school return approach (Maeda et al., 2012a). It targets SR among adolescents (aged 13–15 years) in public junior high schools and it differs substantially from clinic-based CBT with respect to assessment and treatment. Often, school counselors are unable to undertake individual counseling with youth displaying SR (Fujita, 2009) because the youth tend to avoid school when asked by their parents to meet with the counselor there (Maeda, 2012). In Maeda et al.'s (2012a) school-based approach, only the parents visit the school counselor who discusses intervention that does not rely upon assessment and treatment with the young person.

There is preliminary support for this school-based rapid school return approach. Maeda et al. (2012a) reported the case of a 14-year old female who refused to attend school and who threw temper tantrums when her parents tried to get her to attend. Treatment was conducted via the rapid school return approach, including physical escorting by parents, school staff, and the school counselor. Prior to the intervention, the adolescent had spent no time at school for a month. After implementation of the rapid school return approach she attended school 87% of the time and was in class 74% of the time. Maeda (2016) also reported positive results obtained with three adolescents (13–14 years) unwilling to participate in individual treatment sessions for SR. The rapid school return approach was implemented through consultation with the parents and school staff. All three adolescents resumed regular school attendance within a few days of the intervention commencing, although two showed serious resistive responses.

### The Current Study

The increase in SAPs in Japan is likely to include an increase in SR. In turn, more therapists are likely to receive referrals for youth who display SR but are unwilling to participate in individual treatment sessions. In these cases, parents, school staff, and school counselors or psychologists require an alternative means to intervene. The purpose of the current study was to explore the effectiveness of the school-based rapid return approach for adolescents displaying SR. Based on the positive outcomes in case reports of rapid school return (Maeda et al., 2012a; Maeda, 2016) and the poorer outcomes for youth in a wait-list control condition (King et al., 1998), we hypothesized that the rapid school return approach would yield superior response relative to a “wait and see” approach in which parents wait for spontaneous school return. The school-based rapid return was employed with Japanese adolescents refusing to attend school and unwilling to participate in individual treatment sessions offered via psychiatric clinics or child consultation centers. A naturalistic comparison was conducted, whereby outcomes for adolescents whose parents participated in the rapid school return approach were compared to outcomes for adolescents whose parents declined to participate.

## Method

### Participants

Adolescents enrolled in junior high school in the Kyushu area of Japan were eligible for inclusion in the study if parent and teacher reports indicated that the adolescent: (a) met Berg's criteria for SR (Berg, 1997, 2002); (b) had been unwilling to participate in individual sessions for the treatment of SR; (c) had never been diagnosed with a physical or mental disorder; (d) had not been bullied or experienced other interpersonal problems (e.g., quarrels with friends; scolding from teachers); (e) spent most of their time alone during the school day, often watching television, playing video games, surfing the internet, or reading comic books; and (f) did not have concurrent support from other specialists.

Between April 2009 and March 2015 62 cases were identified across five junior high schools (32 males, 30 females; *M* = 13 years, *SD* = 0.8 years; age range = 12–15 years). On average, each adolescent had missed 61 days of school in the current school year. Fourteen of the 62 adolescents (22%) were absent from school more than 100 consecutive school days, 24 adolescents (39%) missed more than 30 days of school (the absence-based criterion in the SAP definition of the Japanese education system), and the other 24 adolescents (39%) were absence for <30 days.

The parents of the 62 adolescents were given the opportunity to implement the school-based rapid school return approach. In 39 cases (19 males and 20 females) the parents agreed to implement the approach. These cases constitute the intervention group. The 39 adolescents were between 12 and 14 years (*M* = 13.4, *SD* = 0.6 years). In 19 of these 39 cases (44%) the families were single-parent families (17 single mothers and 2 single fathers).

The non-intervention group comprised the 23 cases (13 males and 10 females) in which the parents did not agree to implement the approach. The 23 adolescents were aged between 12 and 15 years (*M* = 13.3, *SD* = 1.0 years). Five of these 23 cases (22%) were families with single mothers while the other 18 cases (78%) were two-parent families. There was no age difference between adolescents in the intervention and non-intervention groups [*t*_(60)_ = −0.52, *p* = 0.60] but there was a greater proportion of single-parent families relative to two-parent families in the intervention group [χ^2^_(1,N = 62)_ = 4.44, *p* = 0.04]. Furthermore, there was no difference between the groups with respect to the average number of days absent from school in the current school year [intervention group: *M* = 51, *SD* = 80 days; non-intervention group: *M* = 78, *SD* = 104 days; *t*_(60)_ = −1.1, *p* = 0.26].

### Procedure

The first author (NM) was employed by the local government as a part-time school counselor for 13 junior high schools. In this role he implemented the rapid school return approach within five schools. The approach comprises four main components.

### Introducing the Rapid School Return Approach to Principals of Junior High Schools

To implement the rapid school return approach it was necessary to obtain permission from school principals because they are responsible for the services offered in public compulsory education schools. The first author visited principals at 13 junior high schools to explain the adverse short- and long-term effects of prolonged SR (e.g., academic underachievement; worsening peer relationships; increased risk of social withdrawal and psychiatric illness; family difficulties; future unemployment) and discuss the school-based rapid school return approach for adolescents unwilling to engage in individual treatment sessions. The school counselor and principals also discussed the ethically challenging issue of parents potentially physically escorting their child to school, with the help of school staff as needed. Ultimately, the principals of five junior high schools agreed to implement the approach in their schools in order to address SR. The school counselor asked that each of these principals identify staff in their school who could support the rapid school return approach.

### Selecting a Support Person From Among the School Staff

There were two reasons for selecting a staff member from each school to be the support person at that school. First, it aided the collection of data about the adolescents displaying SR. Second, the support person would be involved in escorting the adolescents to school. The principal prepared a schedule indicating which staff members did not teach during the first period of the day, to identify who could be involved in the escorting process on school mornings. Usually, it was just one person from each school who assisted the parent(s) and school counselor with the cases at that school. Occasionally, other school staff joined this support person when more people were needed to escort the adolescent to school.

### Developing a List of Youth Displaying School Refusal and Collecting Attendance-Related Data

The support staff prepared a list of adolescents who had missed more than four consecutive school days or 10 intermittent school days in the school year so far, and who met Berg's (1997, 2002) SR criteria. For each adolescent on the list, attendance-related data was recorded during the intervention and for at least 9 months thereafter. The attendance-related data included: (a) number of days present/absent; (b) number of classes present/absent, per day; (c) amount of time spent in other special rooms during class-time, such as the school nurse's office; and (d) characteristics of the adolescent's arrival at school from home (i.e., alone, escorted by parents, or escorted by school staff). The recording of this information for at least 9 months after intervention facilitated detection of possible relapse.

### Holding a Support Meeting With the Parents and School Staff

In each case, the school counselor (NM) held a support meeting with the parent(s) of the adolescent, the classroom teacher, the school nurse, and other support staff identified by the principal. In families with two parents, both parents were encouraged to be involved in the meeting.

The two aims of the support meeting were to gather information about the adolescent and to share information about the rapid school return approach. Information about the adolescent was gathered to determine whether the conditions for inclusion were met (see “Participants”). In cases where the conditions were not met, families were referred to other services outside of the school.

Information about a flooding-based rapid school return was presented so that parents could decide if they wanted to implement the approach, and if so, to know how to implement it. After discussing the negative effects of prolonged SR so as to encourage parental involvement (Kearney and Bates, 2005), the school counselor explained the intervention process as follows: (a) at some point after the meeting, the parents would declare to their child that they would be forcing him/her to attend school; (b) the intervention would commence 2 days after the declaration, during which time the parents would encourage their child to get ready for return to school (e.g., preparing textbooks and school uniform); (c) the parents would use planned ignoring of the child's behaviors associated with SR, such as crying, somatic complaints, or tantrums (Heyne and Rollings, 2002); (d) the parents would conceal sharp implements at home to reduce the possibility of self-harming; (e) the parents would wake their child, get him/her changed into the school uniform and escort him/her to the school gate; (f) school staff and the classroom teacher would escort him/her from the school gate to the classroom, perhaps with the support of close friends; (g) school staff would come to the family home if the parents could not escort the adolescent to school on time due to resistive responses (Blagg, 1987); (h) after arriving at school, the adolescent would be expected to stay at school all day (preferably in the classroom for the whole time), and school staff would not permit him/her to leave school early, even if the adolescent wished to do so (Blagg, 1987; Kearney and Bensaheb, 2006); (i) the intervention would be suspended if the parents requested it.

During the support meeting, the school counselor also provided the parents with information about strategies for handling resistive responses (e.g., planned ignoring; escorting by more than two people). The parents and school staff were advised of the likely occurrence of somatic complaints (e.g., stomach ache, reports of feeling unwell) and resistive behaviors (e.g., temper tantrums, verbal abuse, violent behavior, and running away from home or school). Parents and school staff needed to agree that when the adolescent engaged in self-harming behavior or threatened suicide they would stop the intervention and engage in a support meeting during which an appropriate response would be determined (e.g., seeking psychiatric support).

Participating principals and parents gave verbal consent for the intervention to be employed. Parents not consenting to the intervention are those described in the non-intervention group. Written informed consent was not requested because the first author conducted the intervention during the natural course of his work as school counselor.

### Data Analysis

Outcome for adolescents in the intervention and non-intervention groups was based on a treatment response criterion defined as the adolescent achieving at least 85% attendance in the classroom within 3 months and continuing to attend classes at least 85% of the time across the next 6 months. The criterion of 85 percent attendance was based on the Japanese definition for SAPs (i.e., 30 days absence during a school year of 200 days, equivalent to 15% of school time). The amount of attendance during class time was based on a count of the number of classes attended each day, drawn from the records kept by the support person at the school. The proportion of youth in the intervention group who achieved the criterion for treatment response was compared with the proportion of youth in the non-intervention group who achieved that criterion.

## Results

Table 1 presents the outcomes for adolescents in the intervention and non-intervention groups. It includes the proportion achieving full-time attendance (100%) within 1 week or regular attendance (≥85%) within 3 months. It also presents the proportion responding to verbal prompts (e.g., “If you do not go to school with us, we have no choice but to call school staff and ask them to bring you to school”) or physical escorting (i.e., guiding the adolescent by holding their hand or arm; pulling the adolescent into the car or the school building).

**Table 1:** Characteristics of the return to school according to the outcome of intervention.

**Group**	**Number of cases**	**Outcome**	***N***	**Type of intervention**	**Sex**		**Full-time attendance (i.e., 100%) within a week**	**Regular attendance (i.e., ≥85%) within 3 months**
Intervention	39	Response	28	Physical (14)	Male	7	13/14	14/14
					Female	7		
				Verbal (14)	Male	6	12/14	14/14
					Female	8		
		Non-Response	9	Physical (9)	Male	6	4/9	0/9
					Female	3		
				Verbal (0)	Male	0	NA	NA
					Female	0		
		Change of school	2	Physical (1)	Male	0	1/1	NA
					Female	1		
				Verbal (1)	Male	0	0/1	NA
					Female	1		
Non-intervention	23	Response	0	NA	NA		NA	NA
		Non-response	23	NA	Male	13	0/23	0/23
					Female	10		

*Response = 85% attendance in the classroom within 3 months, and at least 85% attendance in the classroom across the next 6 months. NA, Not applicable*.

### Intervention Cases

Twenty-eight of the 39 intervention cases (72%; 13 males and 15 females; $\chi_{\left(1\right)}^{2}$ = 0.14, *p* = 0.70) were classified as treatment responders. That is, the adolescents achieved at least 85% attendance in the classroom within 3 months and for the next 6 months they were in class at least 85% of the time. Of these 28 responders, the majority (*n* = 25) involved a return to full-time school attendance within 1 week after commencement of the intervention. Of the other three responders, two involved a return to full-time school attendance within a month and the other involved return to full-time attendance within 3 months. Half of the 28 responders returned to full-time school attendance in response to verbal prompts for school attendance by parents and school staff. In 12 of these 14 cases the return to school occurred on the first day of intervention. The other 14 treatment response cases involved physical escorting to school, with 13 of the 14 returning to full-time school attendance within 1 week. For those 14 cases only requiring verbal prompting, the average number of days absent from school prior to intervention was 63.7 (*SD* = 104.1), whereas the average number of days absent among the 14 cases requiring physical escorting was 39.1 (*SD* = 68). There was no significant difference between these two groups [*t*_(26)_ = −0.74, *p* = 0.47]. All 14 cases requiring physical escorting showed serious resistive responses when parents and school staff tried to escort them to school (e.g., temper tantrums, screaming, clinging to the bed, a sit-in protest, hiding in the toilet). There were no reports of injury for these 14 adolescents, their parents, or school staff.

Two of the 39 intervention cases (5%; both female) transferred to other schools during the intervention. Outcome data was not available following the change of school. One case involved a change of school due to the strong desire of the adolescent and her family, and the other case involved a change of school for unspecified family reasons.

Nine of the 39 intervention cases (23%; 6 males and 3 females) were classified as non-responders. In five of these cases (4 males and 1 female) the parents and school staff discontinued the escorting process within a month due to the adolescent's serious resistive responses such as violent behavior against parents or school staff, or running away from home and school. There were no reported injuries for these five adolescents, their parents, or school staff. Of the cases in which escorting was discontinued, two adolescents continued to be absent for more than a year, two adolescents were in their final year and consistently refused to attend school until the end of junior high school (~6 months later), and one involved the adolescent attending school mornings at a special education classroom for 3 days a week over 6 months.

The other four non-responders (2 males and 2 females) temporarily achieved regular school attendance following the intervention, but there was relapse to SR within 3 months. Within these 3 months, the parents in all four cases decided to discontinue the rapid school return approach. In one case the mother discontinued without reason, and her child attended school intermittently for a year (54% attendance). In the other three cases the parents stated that they discontinued because other clinicians had advised them to wait until spontaneous recovery occurred. In these three cases, two adolescents persisted in their absenteeism for more than a year and one attended 90 min of special education classes 3 days a week for 7 months.

### Non-intervention Cases

Of the 62 families invited to participate in the rapid return approach, 23 (37%) decided to wait for the adolescent's spontaneous school attendance. In all 23 cases, SR continued for more than 3 months, with none of these adolescents returning to regular class attendance within 9 months. Thus, none of the non-intervention cases fulfilled the criterion for treatment response. Of the 23 cases, 15 involved continued absenteeism for more than a year, 5 involved intermittent attendance in an individual study room at the school, and 3 involved the adolescent attending classes <30% of the time.

## Discussion

There are few reports of treatment for SR when youth refuse to attend therapy. The current study examined the effectiveness of a school-based rapid school return approach for these cases. Following, we discuss uptake of the intervention among Japanese school principals and parents, examine the outcomes, consider cultural influences on the use of rapid school return, present indications and contra-indications for its use, and reflect on the limitations and implications of the study.

### Uptake of the Rapid School Return Approach

Of the 13 principals introduced to the school-based rapid school return approach, five agreed to it being used in their school. They expressed the belief that simply waiting for spontaneous recovery is an unethical practice because it fails to ensure that adolescents engage in compulsory education. The eight principals who did not agree to the approach being used in their school expressed the belief that SR is a family issue which should not be addressed by school staff but via treatment offered in psychiatric clinics or child consultation centers.

Across the five participating schools there were 62 adolescents displaying SR. The parents of 23 adolescents decided to wait until their child attended school again, constituting the non-intervention group. The intervention group comprised 39 cases in which parents agreed to implement the rapid school return approach. The majority of parents in the intervention group expressed the intention to quickly return their child to school even though this could be a burden for them as parents. Moreover, one-third of parents in the intervention group wanted to participate because their child's SR had worsened following advice from staff at a psychiatric clinic or child consultation center to simply wait for spontaneous recovery. It is noteworthy that there was a greater proportion of single-parent families in the intervention group. In Japan, parents who are single (mostly mothers in the current study) may be more likely to accept the rapid school return approach because of a sense that they are struggling to deal with the SR on their own, exacerbating their sense of stress.

### Outcome of the Rapid School Return Approach

Twenty-eight of the 39 intervention cases (72%) were classified as treatment responders, while none of the 23 non-intervention cases returned to full-time school attendance within 9 months, supporting the hypothesis. The fact that there was a greater proportion of single-parent families among the intervention cases discounts the notion that intervention cases were more successful because they more often contained two-parent families.

When parents escort their child to school, somatic complaints, protests, crying, temper tantrums, and negotiation are all likely to occur (Heyne and Rollings, 2002). In the current study, parents and school staff implementing rapid school return were confronted with a variety of resistive responses but they ignored these and persisted in escorting the adolescent to school, in accordance with the school counselor's guidance. This is a crucial aspect of the rapid school return approach. If parents and school staff commence but then discontinue the escorting process, the adolescent's avoidance of school is not eliminated and may actually be reinforced (Maeda et al., 2012a). In 13 of the 14 responder cases that involved physical escorting, parents and school staff reported that the adolescent's resistive responses decreased by the second week of the intervention. It seems that consistency in physical escorting in the face of resistive responses helps ensure that avoidance of school gives way to regular school attendance. The strength of parents' resolve is likely associated with their consistency in physical escorting, and their use of appropriate levels of behavioral control may have helped reduce the intensity and persistence of the adolescent's resistive responses (Smetana, 2017).

Verbal prompts to elicit attendance seem to be sufficient in some cases. Half of the 28 treatment response cases involved a return to full-time school attendance via verbal prompts. That is, parents and school staff firmly stated that they would escort the adolescent to school if they refused to attend of their own accord. In one of these cases the adolescent had been absent from school for 360 school days (one-and-a-half school years).

There was no significant difference between the group of adolescents who responded to verbal prompting and those who responded to physical escorting with respect to the average number of days absent prior to the intervention. It thus seems that the amount of absenteeism prior to intervention is unlikely to be a reliable indicator of the likely outcome of verbal prompting vis-à-vis physical escorting, at least in cases of adolescent SR characterized by the adolescent's unwillingness to participant in therapy sessions. At the same time, it is the clinical impression of the first author (NM) that the 14 cases that responded to verbal prompting shared two features. First, there was no parent-child role reversal. The parents of these adolescents were not observed to have difficulty managing their child's behavior on a daily basis and they did not bow to unreasonable demands from the child (e.g., purchasing expensive gaming software which the adolescent demanded in return for school attendance). The absence of parent-child role reversal is likely to benefit rapid school return because parents often need to adopt a firm attitude toward their child when enforcing school attendance. The second feature observed in cases responding to verbal prompting is that during the support meeting the parents indicated they have good communication with their child. When adolescents are helped to communicate their distress, and when they feel understood, this may reduce their overall level of distress and increase their willingness to attend school (Heyne and Sauter, 2013).

Of the nine non-response cases in the intervention group, four temporarily returned to school following the intervention, but there was relapse to SR within 3 months. The parents decided not to re-apply pressure for school attendance based on the advice of others (e.g., psychiatrists, teachers, friends). The other five non-response cases involved the parents discontinuing intervention following efforts to escort the adolescent to school, because the adolescent's resistive responses were greater than expected (e.g., fleeing from home or school; temper tantrums during the escorting process). It was particularly difficult for these parents to respond to their child's leaving school after classes had started, due to their own work schedules.

There were two commonalities across the nine non-response cases in the intervention group. First, parent-child role reversal was evident. The adolescents in this group regularly made high demands in exchange for school attendance. Moreover, it seemed to be the mothers in these families who accommodated the adolescents' demands. Parent-child role reversal may have rendered the parents less effectual in managing the escorting process. A second and related commonality among most non-response cases was the lack of paternal authority. This has been identified in prior studies of SR, whereby fathers were described as under-involved (Baideme et al., 1979; Blagg, 1987), not holding a responsible parental role (Hersov, 1977), not showing a firm attitude toward the child's school attendance (Kennedy, 1965; Aida, 1978), and needing to become more involved in the escorting process (Ishikawa, 2007; Maeda et al., 2010, 2012b; Maeda, 2012). It has been suggested that fathers are likely to manage adolescents' resistive responses physically and firmly during the process of escorting to school, which may help modify parent-child role reversal between mothers and adolescents (Ishikawa, 2002). At the school, intensive resistive responses from adolescents may necessitate the involvement of more school staff to help parents with escorting (Maeda, 2016), especially when fathers do not actively participate. The involvement of school staff depends on school policy about staff escorting a student into school, which is likely to vary across schools and across cultures.

### Cultural Influences on the Use of a Rapid School Return Approach

The cases presented in this study are not unique to Japan. For example, Blagg (1987) reported on British adolescents who refused school and benefited from a rapid return to school. Those cases were similar to the cases reported in the current study in the following ways: (a) refusal to go to school subsequent to experiences such as school transfer, physical illness, and friendship problems; (b) absence of physical disorder; (c) resistive responses during the escorting process; and (d) lack of paternal authority. One of Blagg's cases involved the treatment of an adolescent male whose mother was overprotective and whose father was uninvolved in managing the SR. The parents had initially chosen for home tuition, which seemed to reinforce the adolescent's SR. Thereafter, the therapist physically escorted the resistive adolescent to school for 2 weeks because the parents seemed incapable of this. The adolescent resumed attendance and was still attending school regularly 1 year later. In the current study, it was sometimes necessary for school staff to help the parents physically escort the adolescent to school. This was decided jointly between the parents and school staff during the initial support meeting, when parents spoke about the difficulty they previously encountered when escorting their child to school.

A difference between Blagg's (1987) intervention and the rapid school return approach reported in the current study is that Blagg used warnings about legal action (e.g., the family being taken to court). The threat of legal action may have been an important factor in the youths' return to school. In Japan it is virtually impossible for school authorities to impose legal sanctions against parents who do not get their children to school (Shinohara, 2008). Although it is permissible by law, the law is rarely applied. Furthermore, local educational boards tend not to put pressure upon parents to pressure their child to attend school (Shinohara, 2008), which may be a result of media coverage about not applying pressure (Kawai and Sakurai, 2003).

A peculiarity of the Japanese education system is that students in compulsory schools can receive automatic promotion to the next year level and a diploma at graduation age regardless of school attendance and individual academic achievement (Maeda and Hatada, 2019). At an individual and family level, this can discourage youth displaying SR and their parents from pursuing regular school attendance. At a community level, it can make it difficult for education and mental health professionals in Japan to value and implement the school-based rapid school return approach.

Clearly, cultural influences will impact the type of SR interventions delivered in different countries, and these influences will also change over time. It is incumbent upon us as education and mental health professionals to discern and deliver culturally and ethically responsive interventions, moving beyond historical and traditional barriers to how we work (Gallardo et al., 2009).

### Indications and Contra-Indications for Rapid School Return

It has long been acknowledged that behavioral intervention involving forced school return can be quite stressful for youth and parents (Blagg and Yule, 1984; Gullone and King, 1991). This may explain, in part, why there have been few examples of behaviorally-oriented rapid school return since the 1990s, the period in which youth-focused CBT for SR became more prominent. Literature published in the 2000s has focused, instead, on the indications and contra-indications associated with rapid return to school for youth displaying SR.

Kearney (2002a) suggested that immediate return to full-time attendance is not preferable when youth have high levels of anxiety and long histories of SR. Wimmer (2003) suggested that the rapid approach be utilized with great caution because of the extreme stress that can be experienced by the people involved. Kearney (2003) advised that rapid school return be stopped when youth become overanxious or parents cannot tolerate it. At the same time, it was pointed out that stopping rapid school return midway reinforces the child's resolve to refuse school.

In a 2004 review of CBT for youth anxiety and depressive disorders, Compton et al. discussed extinction in relation to school phobia. It was argued that “unilateral extinction strategies, such as when a parent returns the school-phobic child to school by force, have significant disadvantages relative to consensual child involvement” (p. 947). Failure to help the young person internalize a strategy for coping with current and future anxiety-provoking situations was held to be a key disadvantage. Another disadvantage reported by Compton et al. is the inability to address symptoms that parents and teachers may not be aware of.

In 2007 Kearney and Albano advocated the following conditions for the use of enforced school attendance: “(a) a child refusing school only for attention and without any significant distress or anxiety; (b) parents who are willing to take a child to school and school officials who are willing to meet the child at the door of the school building and escort her to class; (c) presence of two parents or one parent and another adult who can take the child to school; (d) a child who understands what will happen if she refuses school; (e) a child currently missing most school days; (f) a child under age 11 years” (p. 175). With respect to the first point, education and mental health professionals can use the School Refusal Assessment Scale—Revised (Kearney, 2002b) to assess the prominence of attention-seeking behavior, together with other instruments to assess distress and anxiety (see Ingul et al., 2019). Parsons (2009) also advised that the rapid school return approach only be utilized by specifically trained school counselors.

The youth's developmental level, often estimated via age, is an important consideration for the use of rapid school return. As noted, Kearney and Albano (2007) suggested that rapid school return only be used with youth under 11 years of age. In the current study the approach was used with 39 youth older than 12 years. Almost three quarters of these cases returned to regular attendance at school, calling into question the recommendation of Kearney and Albano. Kennedy's (1965) flooding-based approach was also applied successfully with a group of adolescents (*n* = 13). Despite this, Kennedy did not use the rapid school return approach for Type II SR, characterized in part by being in the “upper grades.” In effect, Kennedy indirectly suggested that the rapid school return approach not be used with older youth displaying SR.

Parent-related factors also warrant consideration when deciding whether to use a rapid school return approach. First, do parents experience psychological difficulties which may impact their role in managing their child's school attendance? Research on exposure-based CBT for youth anxiety suggests that when parents have psychological problems some children may benefit less from parental involvement in treatment (Berman et al., 2000). Psychopathology is frequently observed in the parents of youth displaying SR (Heyne et al., 2015) which may maintain SR if the parents' own anxiety or depression interferes with their capacity to support the child's return to regular schooling. For example, parents may be less effective in their use of instructions and less attentive to any progress made by the young person (Heyne et al., 2004). A second parent-related factor to consider is whether parents are capable of remaining calm and avoiding verbally and physically aggressive behavior when enforcing school attendance. Hostility or conflict between parents and youth needs to be addressed before or during intervention for SR (Kearney and Silverman, 1995), and certainly before intervention involving rapid school return. Close relatives may be called upon if parents lack control or are extremely anxious (Blagg and Yule, 1984) but also if they lack time or the commitment to block their child's avoidance behavior (e.g., Hargett and Webster, 1996). Third, do the parents believe they are able to enforce school attendance? Even if parents are able to regulate their emotions, uncertainty about whether they are able to implement the rapid return approach would likely impede the procedure. Parent self-efficacy can be measured via the Self-Efficacy Questionnaire for Responding to School Attendance Problems (Heyne et al., 2007).

The few accounts of rapid school return that have been reported since the 1990s emerge from Japan (e.g., Sonoda and Takayama, 2006; Ishikawa, 2007; Maeda, 2011, 2012, 2016; Maeda et al., 2012b). Contra-indications reported in these studies relate to the presence of physical or mental disorders for youth and the experience of bullying at school. The main indication for considering rapid school return was the youth's unwillingness to participate in therapy sessions.

In the absence of robust empirically-derived guidelines, education and mental health professionals must weigh up the relative merits of a flooding approach versus a part-time increase in school attendance. According to Yule et al. (1980), each approach can work in particular cases and “the problem is to know before-hand which approach to try first with which cases” (p. 276). King and Ollendick (1989) reviewed desensitization-based gradual school return and flooding-based rapid school return as behavioral interventions for SR. They argued that rapid school return would help minimize secondary gain (e.g., the child enjoys watching television when not at school) while gradual return would be required for school refusers with severe anxiety who are uncomfortable with the rapid approach. Indeed, the severe and chronic cases of SR, conceptualized as Tier 3 cases in the Kearney and Graczyk (2014) response-to-intervention model for absenteeism, likely warrant more intensive assessment and graduated school return, relative to Tier 2 cases of emerging SR. However, if it is not possible for the therapist to meet with the young person to conduct assessment and treatment, a parent-focused flooding-based approach may need to be considered, in view of the negative outcomes associated with continued absence from school.

Summarizing the various considerations about this approach, rapid school return may be indicated when: (a) youth cannot be encouraged to participate in treatment (Maeda, 2016); (b) they do not have genuine physical problems (Blagg and Yule, 1984) or serious mental health problems (Rodriguez et al., 1959); (c) they are not overly anxious (King and Ollendick, 1989; Kearney, 2002a; Kearney and Albano, 2007) and have not experienced bullying at school (Ishikawa, 2007; Maeda, 2011, 2012) (d) the young person is enrolled at an appropriate school (Blagg and Yule, 1984); (e) parents and school staff agree on the use of rapid return and school staff can make arrangements to help the young person settle in at school (Blagg and Yule, 1984; Kearney and Albano, 2007), such as opportunities to meet with preferred teachers without this becoming an avoidance of class time (Peterman et al., 2015); (f) two parents or other appropriate support people can be involved in escorting (Blagg and Yule, 1984; Kearney and Albano, 2007); (g) parents receive detailed advice about how to respond to the young person's resistive behavior (Blagg and Yule, 1984); and (h) school staff receive adequate training in the use of the approach (Parsons, 2009). Rapid return is contra-indicated when parents experience difficulties (e.g., anger management; depression; low self-efficacy) and there are no substitute support people available to escort the young person to school.

Extrapolating from Compton et al. (2004), once the young person is attending school again, arrangements should be made to assess his or her social-emotional functioning and build coping skills. As an example, Maeda (2012) reported that seven sessions of social skills training were offered to a young person who resumed school attendance following implementation of the school-based rapid school return approach. Similarly, family dynamics should not be ignored simply because a decision is made to employ rapid school return under appropriate conditions. Once youth are attending school again, attention can shift to the parents' role in granting appropriate autonomy to their adolescent child. For example, in a treatment for anorexia nervosa in adolescents, parents are initially responsible for supervising aspects of the intervention (e.g., eating behavior) while in later phases parents reduce their authority and “take a step back” (Le Grange et al., 2005). Parents are then encouraged to engage in discussions with the adolescent about the adolescent increasing personal autonomy and the parent decreasing authority in areas of the adolescent's life.

### Limitations and Further Research

The current study has limitations. First, no assessment was undertaken with the adolescents because of their refusal to attend sessions with the counselor. Thus, there was no pre- or post-intervention data on the adolescents' mental health status (except pre-intervention information from parents and teachers about the absence of diagnosed mental disorders) or severity of SR (except for data on absenteeism). Thus, the short- and long-term social-emotional benefits of school return are unknown. Second, this study was an uncontrolled case series. Randomized controlled trials need to be conducted to establish the effectiveness of the rapid school return approach. A wait-list control condition might be judged unethical by school principals in Japanese compulsory schools, necessitating a comparison with treatment as usual and matching youth on key variables (e.g., age, gender, length of SR). Controlled case studies present another option, incorporating regular measurement of the adolescent's social-emotional functioning. Factors that potentially moderate the outcome of rapid school return should be measured in future trials (e.g., family functioning; parenting styles and dimensions; youth temperament) along with treatment acceptability for youth, parents, and school staff.

## Conclusion

The current study explored the effectiveness of a school-based rapid school return approach for adolescents displaying SR. The approach is implemented via the school counselor with parents and school staff; no individual sessions are conducted with the young person. The case series presented here preliminarily suggests that positive results can be achieved for a sizable group of adolescents who display SR and are unwilling to come to individual therapy sessions. The results also suggest that waiting for the adolescent's spontaneous school attendance may best be avoided. The extent to which adolescents engage in resistive responses when being escorted to school may be associated with parenting factors such as parent-child role reversal and diminished paternal authority. These factors, as well as the other indications and contra-indications presented here, should be carefully assessed during the support meeting with parents, prior to implementing rapid school return. Robust evidence for the effectiveness of the rapid school return approach with adolescents is yet to be garnered. The conditions needed to ensure optimal short-term and long-term outcomes for adolescents also need to be investigated.

## Data Availability Statement

Requests to access the datasets should be directed to Naoki Maeda: naoki225@phoenix.ac.jp

## Ethics Statement

Ethical review and approval was not required for the study in accordance with local legislation and institutional requirements. Written informed consent for participation was not provided by the participants' legal guardians/next of kin because this first author conducted the intervention described in this manuscript during the course of his work as school counselor. The intervention was not conducted in the context of a research project. The school principals and parents gave verbal consent for the intervention to be employed. Parents not consenting to the intervention are those referenced as the non-intervention group.

## Author Contributions

NM designed the study and was responsible for the intervention, data collection, and data analysis. DH contributed to the conceptualization and writing of the manuscript, with major contributions to the Introduction and Discussion. NM and DH wrote sections of the manuscript, contributed to manuscript revision, and read and approved the submitted version.

### Conflict of Interest

The authors declare that the research was conducted in the absence of any commercial or financial relationships that could be construed as a potential conflict of interest.

## Footnotes

^1^The term ‘youth' is used to refer to children and adolescents.
